# A Table, Shewing the Different Diseases and Casualties Which Occurred on Board His Majesty's Ship, Astræa, from Her Leaving England for the Jamaica Station, in January, 1787, to Her Arrival at Chatham, in June, 1790; Comprehending a Period of Three Years and Six Months

**Published:** 1799-06

**Authors:** 


A TABLE, *ſhewing the different Diſeaſes and Caſualties which occurred on board his Majeſty's Ship, Aſtræa, from her leaving England for the Jamaica Station, in January, 1787, to her Arrival at Chatham, in June, 1790; comprehending a Period of three Years and ſix Months.*

| DISEASES. | |
|---|---|
| Pulmonic complaints | 5 |
| Rheumatiſm | 2 |
| Remittent fever | 68 |
| Intermittent fever | 52 |
| Slight fever, the effect of cold, or irregularities | 97 |
| Dyſentery | 7 |
| Diarrhœa | 25 |
| Hepatic complaints | 15 |
| Spaſmodic affections | 31 |
| Scurvy | 4 |
| Epidemic catarrh | 50 |
| Ulcers | 12 |
| Contuſions and ſlight wounds | 372 |
| Fractures | 6 |
| Number of Patients | 741 |

| | |
|---|---|
| Sent to the hoſpital | 111 |
| Invalided to England | 11 |
| Died in hoſpital | 8 |
| Died on board | none |

| | Years. | Months. | Days. |
|---|---|---|---|
| At Sea, or in Ports where no ſick were landed | 1 | 2 | 26 |
| At Port-Royal | 1 | 4 | 5 |
| In Kingſton Harbour | 0 | 9 | 4 |
| Ports in England | 0 | 2 | 0 |

Of the deaths in the hoſpital, three were from accidents, and one a marine who was ill of a pulmonary complaint, ſix months before he left England; ſo that four can only be ſaid to have died from diſeaſes of the climate.

Of the eleven invalided, or ſent to England for change of climate, five were received on board, at Jamaica, labouring under chronic complaints, contracted on ſhore:—Capt. Rainier very humanely received them for that benevolent purpoſe. From the nature of their diſorders they had no chance of recovering while they remained in the country.

